# Differential aspects of attention predict the depth of visual working memory encoding: Evidence from pupillometry

**DOI:** 10.1167/jov.23.6.9

**Published:** 2023-06-15

**Authors:** Damian Koevoet, Marnix Naber, Christoph Strauch, Rosyl S. Somai, Stefan Van der Stigchel

**Affiliations:** 1 Experimental Psychology, Helmholtz Institute, Utrecht University, Utrecht, The Netherlands; 2 Experimental Psychology, Helmholtz Institute, Utrecht University, Utrecht, The Netherlands; 3 Experimental Psychology, Helmholtz Institute, Utrecht University, Utrecht, The Netherlands; 4 Psychology, Faculty of Natural Sciences, University of Stirling, Stirling, UK; 5 Experimental Psychology, Helmholtz Institute, Utrecht University, Utrecht, The Netherlands

**Keywords:** visual working memory encoding, pupillometry, orienting, alerting, precision, internal-external memory trade-off

## Abstract

What determines how much one encodes into visual working memory? Traditionally, encoding depth is considered to be indexed by spatiotemporal properties of gaze, such as gaze position and dwell time. Although these properties inform about *where* and *how long* one looks, they do not necessarily inform about the current arousal state or *how strongly* attention is deployed to facilitate encoding. Here, we found that two types of pupillary dynamics predict how much information is encoded during a copy task. The task involved encoding a spatial pattern of multiple items for later reproduction. Results showed that smaller baseline pupil sizes preceding and stronger pupil orienting responses during encoding predicted that more information was encoded into visual working memory. Additionally, we show that pupil size reflects not only how much but also how precisely material is encoded. We argue that a smaller pupil size preceding encoding is related to increased exploitation, whereas larger pupil constrictions signal stronger attentional (re)orienting to the to-be-encoded pattern. Our findings support the notion that the depth of visual working memory encoding is the integrative outcome of differential aspects of attention: how alert one is, how much attention one deploys, and how long it is deployed. Together, these factors determine how much information is encoded into visual working memory.

## Introduction

Visual working memory (VWM) allows one to briefly maintain and manipulate visual information. Fundamental for flexible and intelligent behavior, VWM has been of great interest within the field of (cognitive) neuroscience and psychology. When given the option, participants look at stimuli longer whenever visual processing is more difficult (Meghanathan, van Leeuwen, & Nikolaev, 2015; Peterson, Beck, & Wong, 2008). In the context of VWM, it is often implied that fixation duration on stimuli (directly) reflects encoding depth (e.g., Draschkow, Kallmayer, & Nobre, 2021; Somai, Schut, & Van der Stigchel, 2020). Although gaze position and dwell time may reveal *where* and *how long* participants look, they do not reveal all aspects of *how strongly* attention is deployed. For example, longer dwell times do not necessarily index deeper processing of the fixated material (Schad, Nuthmann, & Engbert, 2012). Because pupil size may capture several overlapping, yet independent, aspects of attention (Strauch, Wang, Einhäuser, Van der Stigchel, & Naber, 2022), here we propose that pupil size may capture how strongly attention is deployed during VWM encoding.

One promising pupillary signal that could capture the degree of encoding is the pupil orienting response to incoming stimuli. The pupil orienting response is a constriction that starts around 200 to 300 ms and ends around 700 to 1200 ms after stimulus onset. The constriction amplitude scales with stimulus relevance (or salience; conspicuity) and has been described as a marker for the *depth of sensory processing* (Binda & Gamlin, 2017; Mathôt & Van der Stigchel, 2015; Naber, Frässle, Rutishauser, & Einhäuser, 2013; Strauch et al., 2022). The pupil light response, which coincides with the orienting response, is indicative of the encoding strength of differently bright stimuli (Blom, Mathôt, Olivers, & Van der Stigchel, 2016; Zokaei, Board, Manohar, & Nobre, 2019). However, stronger attentional orienting should capture encoding strength even regardless of brightness, as has been demonstrated for long-term memory (Naber, Frässle, et al., 2013). Another relevant factor beyond orienting is alerting, which is also captured by pupillary dynamics in the form of baseline pupil size that reflects tonic locus coeruleus (LC) firing (Aston-Jones & Cohen, 2005; Gilzenrat, Nieuwenhuis, Jepma, & Cohen, 2010; Jepma & Nieuwenhuis, 2011; Joshi, Li, Kalwani, & Gold, 2016; Naber & Murphy, 2020). Following the prominent adaptive gain theory (Aston-Jones & Cohen, 2005), the relationship between tonic LC firing (and therefore baseline pupil size) and task performance follows an inverted-U relationship. In this framework, very low and very high tonic LC firing rates are indicative of drowsiness and exploration/stress, respectively. In contrast, moderate LC firing rate is linked to optimal task performance. It is difficult to predict “where” any data point is positioned along this inverted-U in cases when participants do not necessarily get drowsy or stressed during a task. Nevertheless, due to the link between baseline pupil size and performance on tasks that require attention (Aston-Jones & Cohen, 2005; Gilzenrat et al., 2010; Jepma & Nieuwenhuis, 2011), we expected that baseline pupil size could be linked to VWM encoding. More specifically, we expected that smaller baseline pupil sizes accompany deeper VWM encoding in relatively difficult tasks because participants are likely on the right side of the inverted-U curve (toward over-arousal). This prediction would reverse in simpler tasks, as participants would be generally under-aroused and thus on the left side of the curve.

To compare how dwell times and pupil signal components predict how much information is encoded into VWM, paradigms are needed that let participants decide on how long and how intensely to encode themselves rather than presenting to-be-encoded items just once for a fixed amount of time (as in Ballard, Hayhoe, & Pelz, 1995; Draschkow et al., 2021; Melnik, Schüler, Rothkopf, & König, 2018; Somai et al., 2020). One such paradigm is the copy task, wherein participants encode a pattern of stimuli and subsequently rebuild this pattern elsewhere. The availability of to-be-copied items is often manipulated in such tasks. When items remain available externally, it is arguably beneficial to simply look at the information again instead of internally storing it (O'Regan, 1992; Risko & Gilbert, 2016; Van der Stigchel, 2020). Indeed, accumulating evidence suggests that VWM is likely used sparsely if task-relevant visual information remains available in the external world (Ballard et al., 1995; Draschkow et al., 2021; Melnik et al., 2018; Sahakian, Gayet, Paffen, & Van der Stigchel, 2023; Somai et al., 2020). Such paradigms thus provoke shifts in encoding strategy between conditions. Somai et al. (2020) employed a copy task in which the to-be-copied visual information could be resampled from the environment. The authors investigated whether participants would opt to store information in VWM or whether they would choose to resample external information by making a saccade. To investigate which factors drive resampling behavior, the cost to sample from the external world was manipulated by implementing a variable delay time that had to pass until the external information could be (re-)accessed (200-, 1500-, and 3000-ms delays). Participants sampled external information more often when the cost (in delay time) of sampling was low but shifted more toward storing internally when sampling costs were increased. Together, these studies argue for a continuous trade-off between external sampling and internal storage that characterizes natural VWM usage (Ballard et al., 1995; Draschkow et al., 2021; Melnik et al., 2018; Somai et al., 2020). However, VWM encoding depth was assessed using only dwell times, which does not inform about all aspects of how attention is deployed. Therefore, here we explore novel pupillometric indicators of encoding strength.

We reanalyzed data from Somai et al. (2020) with an emphasis on pupillometry. These data were optimal for the current analyses on the link between pupil size and VWM encoding, as participants shifted their encoding strategy between conditions. Based on the *depth of sensory processing* account, we hypothesized that stronger pupil orienting responses are linked to deeper VWM encoding. In line with the adaptive gain theory, small baseline pupil size was hypothesized to be associated with encoding more information, as the copy task is a relatively demanding task (i.e., remembering and reproducing six items).

## Methods

### Procedure

**Figure 1.**
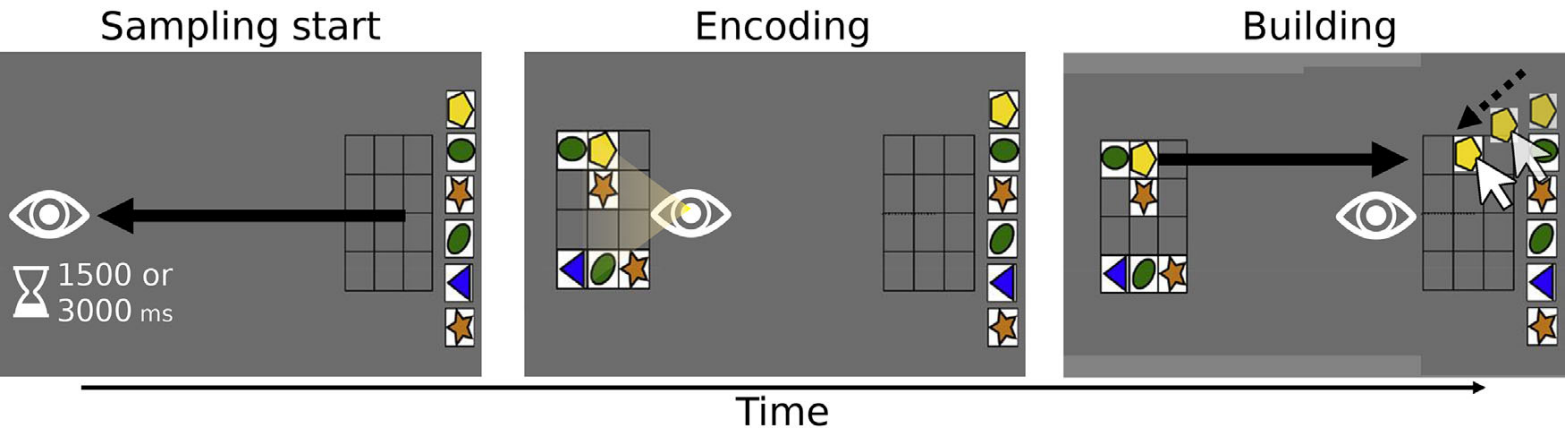
Schematic overview of different phases within a single sampling event. Sampling events start whenever participants look at the left side of the display (eye and yellow gradient indicate gaze). Whenever participants held their gaze position at the left side of the screen for the entire delay duration (1500 and 3000 ms in the medium and long conditions, respectively), the model was presented. Upon model onset, participants could encode information into visual working memory. Subsequently, participants built the model in the response grid on the right using the mouse (see dashed arrow). After building, participants could start the next sampling event. When all six items had been placed in the response grid, the trial ended (regardless of correct/incorrect placements). (Adapted from Somai et al., 2020.)

In total 24 participants took part in the two experiments reported in Somai et al. (2020). The experiments were identical apart from the stimuli that were used; colored shapes (see Figure 1) and non-verbalizable shapes (Arnoult, 1956) were used in Experiments 1 and 2, respectively. On every trial, participants had to copy a pattern of six stimuli presented on the left side of the display. To copy the pattern, participants dragged items using the mouse to the response grid on the right side of the display. Participants were free to look at the to-be-copied pattern as often and as long as they wanted. However, to introduce a cost associated with sampling the to-be-copied pattern, a varying time delay was introduced. In the short, medium, and long delay conditions participants had to wait 200, 1500, or 3000 ms before they could sample the to-be-copied information. Generally, longer delay conditions led to less sampling and were accompanied with more and longer encoding into VWM (Somai et al., 2020). Participants completed 35 trials in each condition.

### Data processing

All data processing and analyses were performed in Python 3.9.7 (Python Software Foundation, Wilmington, DE) and R 4.0.3 (R Foundation for Statistical Computing, Vienna, Austria). All data and scripts necessary to perform the analyses here are openly available at https://osf.io/ckty7/. Pupil size data were analyzed in line with recommendations from Strauch et al. (2022). Pupil size and gaze position data were recorded from the left eye using an EyeLink 1000 with a sample rate of 1000 Hz (SR Research, Mississauga, ON, Canada). Only trials in which all items were placed correctly were included in the analyses (11.37% of the trials were excluded). Because dwell times were too short to allow for reliable pupil size estimates in the short condition, only trials from the medium and long wait conditions were included in the current analyses (see below).

Pupil size metrics and dwell time were computed per sampling event using the following procedure. First, a sampling event was defined whenever participants looked at the left half of the display until the model was presented (see Figure 1). Second, dwell time was calculated as the time until gaze position shifted (back) toward the right side of the display for at least 100 ms. Third, because behavioral data concerning item placements per sampling event (and thus intermittent placement accuracy) were unavailable in the current data, we used build duration as a proxy for encoding depth. For the current analysis, build duration was calculated by determining the time participants remained on the right side of the screen after dwell time ended. Build durations ended whenever gaze position shifted (back) toward the left side of the display for 100 ms consecutively or if the trial ended after building (i.e., whenever the sixth item was placed). In total, valid dwell times and build durations could be calculated for 2778 sample events. Importantly, the number of correctly placed items in the response grid for a given sampling event strongly correlated with build duration, as we verified with open data from Sahakian et al. (2023) (ρ = 0.89, *p* < 0.001; see Supplementary Materials for more details). It should be noted that, whenever incorrect placements are also considered, build duration is associated with incorrect placements and not with correct placements. This means that build duration is not a valid measure of encoding depth in every single situation. Nevertheless, the correlation between correct placements and build duration supports the notion that longer build durations were strongly associated with encoding more visual information in the current data, in which no incorrect placements occurred.

**Figure 2.**
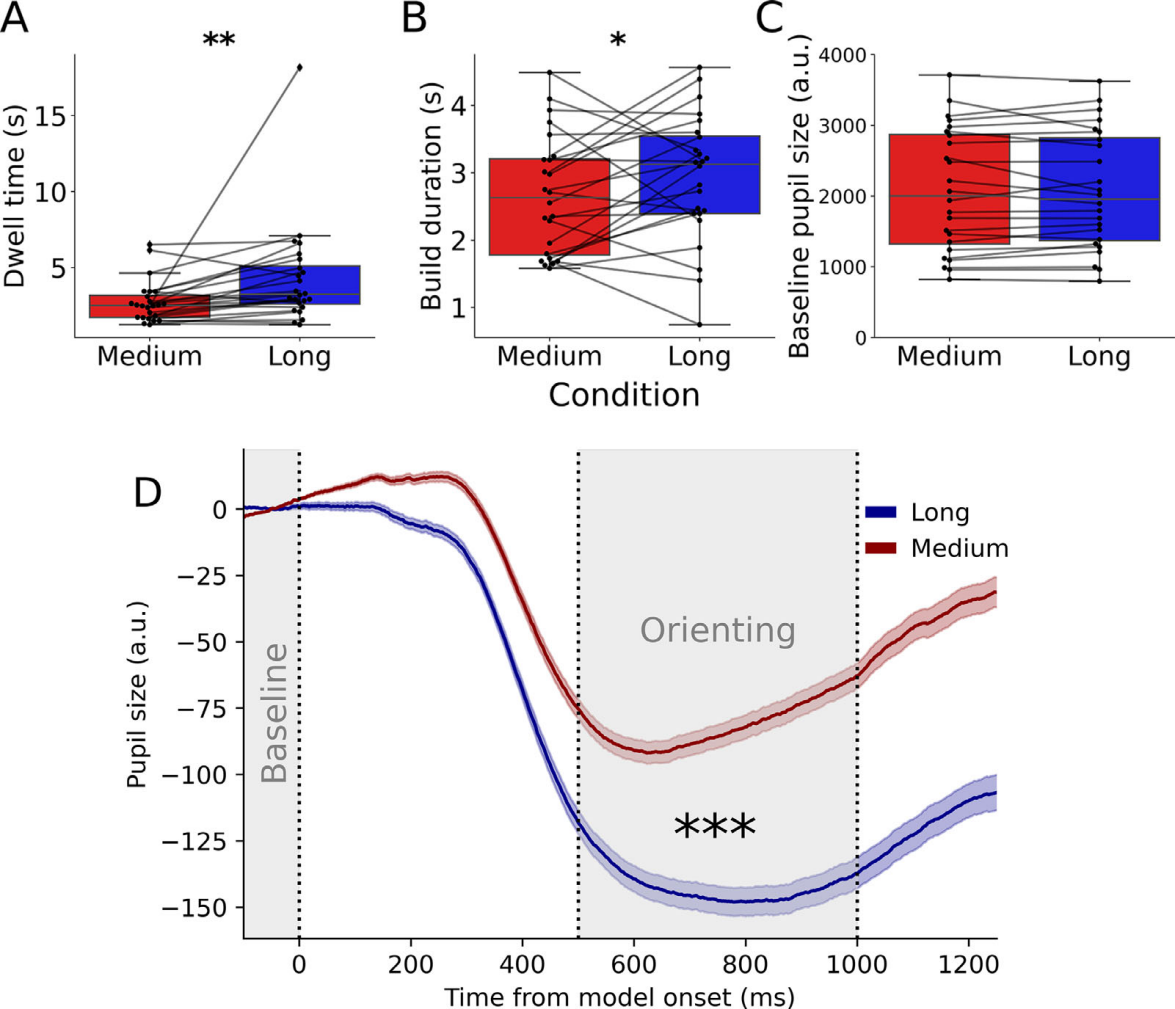
Overview of differences between the two delay conditions (medium = 1500 ms; long = 3000 ms). (A–C) Boxplots showing the differences in dwell time, build duration, and baseline pupil size between conditions collapsed across both experiments. Black dots represent mean values per participant. Lines are drawn to visualize within-subject differences. (D) Pupillary responses between the two conditions collapsed across experiments. The light gray shadings indicate where pupil response components were selected. In the first shading, the median pupil size was computed as a measure of baseline pupil size. The median pupil size in the second shading was used to calculate the amplitude of the orienting constriction response. *p < 0.05, **p < 0.01, ***p < 0.001.

For every sampling event we also determined baseline pupil size and the amplitude of the pupil orienting response. Baseline pupil size was calculated by using the median pupil size 100 ms before model onset (–100 to 0 ms) (Figure 2A). Before calculating orienting amplitudes, pupil size data were subtractively baseline corrected using baseline pupil sizes. Subsequently, orienting response amplitudes were calculated by calculating the median pupil size between 500 and 1000 ms after model onset. To ensure that our pupil size estimates were not affected by gaze angle errors, only sampling events during which participants looked at the model grid (up to 2° deviation from the grid) for at least 1000 ms after model onset were considered (57.34% of sample events included; *M* = 66.38, *SD* = 22.35 samples events per participant); this is also why the short condition was omitted from the current analyses. Note that, due to relatively short periods without eye movements in the data, it was not feasible to analyze an effort-related pupil dilation response because this response takes 2 to 3 seconds to occur (Beatty, 1982; Strauch et al., 2022).

### Data analysis

For statistical analyses, linear mixed-effects (LME) models were used with a significance threshold of *t* > 1.96, corresponding to α = 0.05. Models were selected using recommendations from Barr (2013), methodological grounds, and Akaike information criterion (AIC)-based selection. We modeled the random slopes for the variable delay condition in every LME model to limit type 1 errors (Barr, 2013).

First, we investigated differences among the delay conditions in dwell time, build duration, baseline pupil size, and orienting response amplitudes. These models included both main effects of delay Condition and Experiment, with an R formula of Outcome ∼ Condition + Experiment + (1 + Condition|Participant). Based on AIC selection for all models, the interaction term between Condition and Experiment was not included in these analyses.

Next, we tested how the ocular metrics predicted build duration on a sample-by-sample event basis. To account for possible differences between experiments, we added all interaction terms between Experiment and the ocular metrics; AIC-based model selection also indicated that including these terms significantly improved the fit (see Supplementary Materials). We also included Condition as a covariate in the model to determine whether potential effects were driven by the different variable delay conditions, with an R formula of Build duration ∼ Baseline pupil size x Experiment + Orienting response × Experiment + Dwell time × Experiment + Condition + (1 + Condition|Participant). Whenever interaction terms were found to be significant, these were explored further with separate LME models per experiment, with an R formula of Build duration ∼ Baseline pupil size + Orienting response + Dwell time + Condition + (1 + Condition|Participant).

## Results

### Orienting and dwell time reveal encoding strategy

Somai et al. (2020) reported that participants shifted strategies between the medium and long conditions. More specifically, participants sampled the model grid less often, but sampled longer when the cost of accessing external information was higher (β = 1.64 ± 0.57, *t* = 2.88, *p* = 0.008) (Figure 2A). Here, we extended this finding by also showing that build durations were longer when sampling costs were high (β = 0.33 ± 0.15, *t* = 2.09, *p* = 0.048) (Figure 2B). Neither build duration nor dwell time differed between experiments significantly (*t* < 0.55, *p* > 0.59). Together, this reflected a shift toward deeper or more encoding in VWM when time is at stake due to increased delays during resampling efforts. Next, we investigated whether the pupil orienting response and baseline pupil size differed among conditions and were therefore linked to shifts in encoding strategy (Figure 2).

Baseline pupil size did not differ between conditions (β = 8.97 ± 35.49, *t* = 0.25, *p* = 0.800), implying that the shift in strategy was not reflected in baseline arousal levels (Figure 2C). However, baseline pupil size was larger in the second experiment, which was likely due to the darker stimulus material used in this experiment, leading to dilation of the pupil (β = 1312.68 ± 214.87, *t* = 6.11, *p* < 0.001). The pupil orienting constriction response (Figure 2D) was more pronounced in the long delay condition than in the medium delay condition (β = 59.53 ± 10.79, *t* = 5.52, *p* < 0.001) and did not differ significantly between experiments (β = 23.44 ± 28.38, *t* = 0.83, *p* = 0.417). Note that the initial (small) dilation around ∼50 to 200 ms after model onset can only be driven by factors occurring before model onset because the pupil reacts to changes in visual input with a latency of at least 200 ms. Control analyses in which orienting responses were calculated by subtracting the maximum pupil size 0 to 300 ms after cue onset from the minimal pupil size in the 500- to 1000-ms window show that this limited dilation did not drive the difference in orienting response amplitudes between conditions or the other results (analysis not reported). Already within 500 to 1000 ms after model onset, up to several seconds before the actual building commenced, the pupil orienting response revealed a shift in strategy between conditions, where the intensity of attention was adjusted dynamically.

### Pupil orienting, baseline pupil size, and dwell time predict encoding depth across sample events

In addition to the differences between conditions, naturally, differences between trials—and over sampling events—were apparent. Next, we asked whether fluctuations in encoding depth (build duration) across and within trials could be predicted from the preceding pupil orienting response, baseline pupil size, and dwell time. An LME model collapsed across data of both experiments was fit to investigate this, with an R formula of Build duration ∼ Baseline pupil size × Experiment + Orienting response × Experiment + Dwell time × Experiment + Condition + (1 + Condition|Participant).

**Figure 3.**
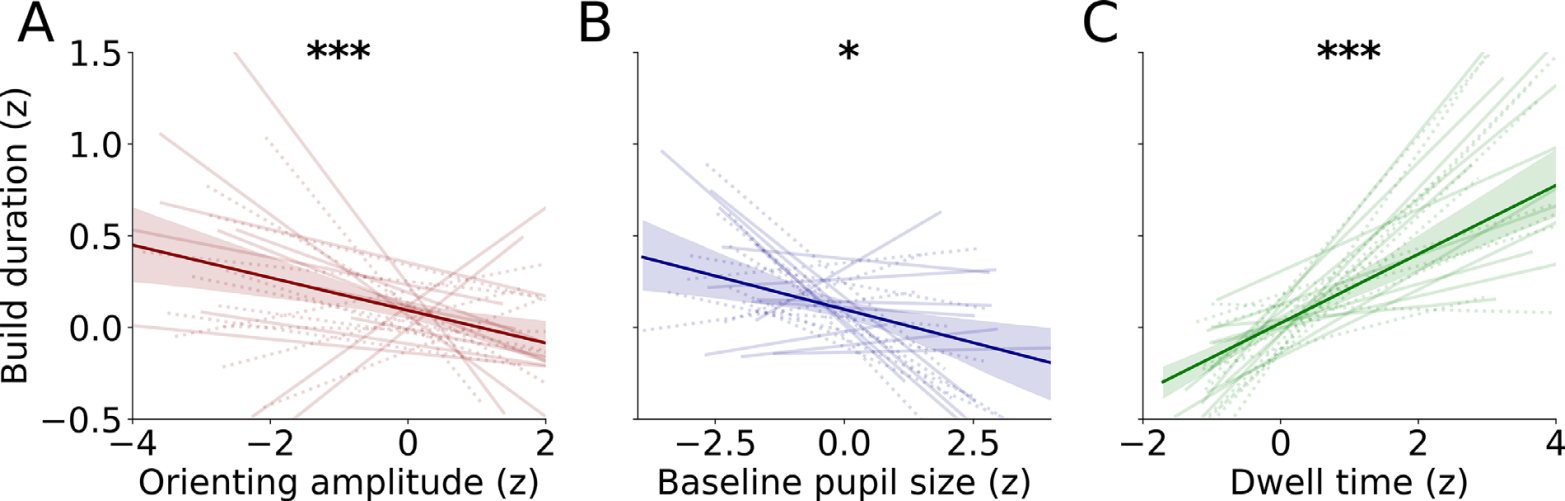
Ocular metrics predict encoding depth. (A) Pupil orienting amplitude, (B) baseline pupil size, and (C) dwell time predict build duration. Transparent lines are linear regression fits to data per participant. Thick lines show the relationship between the ocular metrics and build duration pooled over all trials (error bars reflect bootstrapped 95% confidence intervals). Transparent lines indicate individual participants. Solid lines and dashed lines indicate Experiments 1 and 2, respectively. For visualization, robust *z*-scores are plotted (non-transformed plots can be found in the Supplementary Materials). Robust *z*-scores were computed by subtracting the median and subsequently dividing by the median absolute deviation for each variable per participant (see Rousseeuw & Hubert, 2011). *p < 0.05, ***p < 0.001.

The LME model showed that the pupil orienting response (β = 0.003 ± 0.0001, *t* = 4.04, *p* < 0.001), baseline pupil size (β = 0.0009 ± 0.0003, *t* = 2.53, *p* = 0.014), and dwell time (β = 0.10 ± 0.01, *t* = 7.29, *p* < 0.001) significantly predicted the subsequent building duration (Figure 3). Notably, Condition did not significantly predict build duration in this model, meaning that these effects were not driven by the differences in delay time (β = 0.04 ± 0.12, *t* = 0.34, *p* = 0.736). These findings show that stronger orienting responses predicted longer build durations. This relatively fast (and often ignored) pupil response thus revealed how much one will encode into VWM, which is in line with the *depth of sensory processing* account (Binda & Gamlin, 2017; Mathôt & Van der Stigchel, 2015; Strauch et al., 2022). For the first time, to the best of our knowledge, we are reporting a physiological marker (namely, the pupil orienting response) that reveals changes in the depth of encoding during a shift from external sampling to internal storing. Even preceding the pupil orienting response, a relatively small baseline pupil size significantly predicted longer build durations, seconds before building even commences. This finding is potentially compatible with the adaptive gain theory (Aston-Jones & Cohen, 2005), wherein tonic LC firing and thus baseline pupil size are linked to task performance. When baseline pupil sizes were relatively small, this approached the “peak” of the inverted-U, whereas participants may have been highly aroused whenever their pupils were relatively large during baseline, leading to worse performance. Because the copy task is a quite effortful task (i.e., remembering and placing six items) (see Robison & Unsworth, 2019), it is likely that the arousal levels of participants were indeed positioned on this “right” side of the inverted-U curve. Finally, in line with previous work, longer dwell times were linked with storing more visual information in VWM (Draschkow et al., 2021; Somai et al., 2020). Together, these results show that ocular metrics reflect not only *where* and *how long* attention is deployed but also *how strongly* it is deployed, as well as the current attentional state, and together these attentional aspects determine how much is encoded into VWM.

Next, we examined the interaction terms to determine possible differences between experiments (see Figure 3). The link between dwell times and build duration was even stronger in Experiment 2 than in Experiment 1, as evidenced by the interaction term (β = 0.09 ± 0.03, *t* = 3.54, *p* < 0.001). Dwell time remained a significant predictor in both experiments (Experiment 1: β = 0.10 ± 0.01, *t* = 6.67, *p* < 0.001; Experiment 2: β = 0.19 ± 0.02, *t* = 9.83, *p* < 0.001). The interaction between orienting and experiment was also significant (β = 0.003 ± 0.0008, *t* = 3.33, *p* < 0.001), meaning that orienting predicted building durations differently well across experiments (Experiment 1: β = 0.003 ± 0.0008, *t* = 3.91, *p* < 0.001; Experiment 2: β = 0.0001 ± 0.0003, *t* = 0.41, *p* = 0.683). Although not significant for Experiment 2 in isolation, orienting amplitudes did significantly predict build duration as a main effect collapsed across both experiments, arguing for a relationship between the two. Together, this argues in favor of a predictive effect of the pupil orienting response amplitude on build duration. The main effect of experiment as well as the interaction between baseline pupil size and experiment were not significant (*t* < 1.3, *p* > 0.21), indicating that the predictive effect of baseline pupil size on build duration did not differ between experiments and that build duration in general did not differ between experiments.

### Pupillary dynamics predict encoding precision

The analyses described above show clear links between pupillary dynamics and build duration, which is a proxy for the number of encoded items. Baseline pupil size and the pupil orienting response are thought to reflect the depth of encoding and therefore not only the number but also the *precision* of what is encoded that should be captured by these pupillary components. To address this directly, we reanalyzed data from Zhou, Lorist, and Mathôt (2022). We first provide a summary of the task and results from the original paper and subsequently describe our two novel analyses.

Briefly, participants remembered prototypical or non-prototypical colors (e.g., blue or between blue and green) at differing set sizes (one, two, three, or four colors) and ultimately reported the hue of a memorized color on a color wheel. Zhou et al. (2022) showed an interaction between set size and prototypicality on pupil size. More specifically, with increasing set sizes participants relied more on categorical representations for non-prototypical colors. Participants likely stored categorical instead of continuous representations at the highest set size to lower the effort necessary to complete the task (at the cost of precision). Here, we instead assessed whether baseline pupil size and the pupil orienting response can predict the precision of VWM encoding. To this end, we conducted two lines of analysis. First, we investigated if and *when* precision was reflected in the pupil. Second, we adopted a similar approach as in the main analyses and tested whether pupillary dynamics predict the precision of encoding on a trial-by-trial basis.

**Figure 4.**
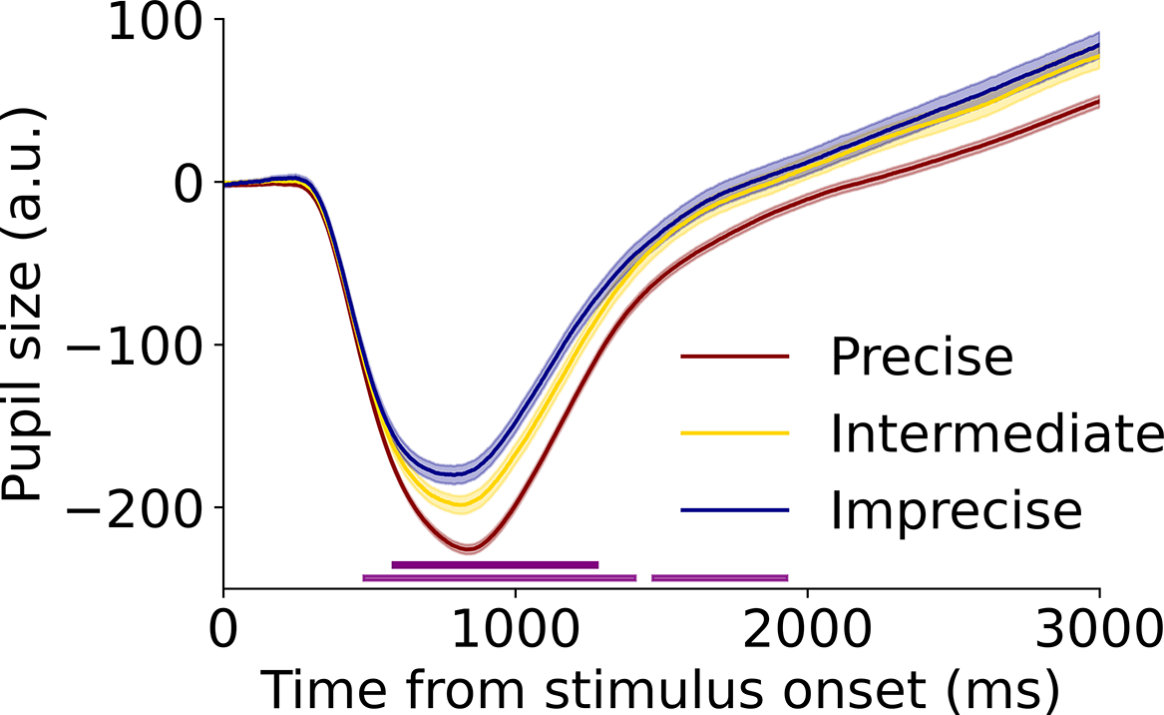
Pupil size reveals the precision of VWM encoding. Error bars reflect the standard error of the mean. Horizontal lines at the bottom indicate significant differences. Dark purple and light purple reflect *p* < 0.01 and *p* < 0.05, respectively.

To determine whether and if so *when* the pupil captures the precision of VWM encoding, we analyzed pupil size over time. If the pupil would capture how precisely items are encoded into VWM, pupil size should differ depending on how accurate participants responded at the end of a trial. Trials (7267 in total) were split into three precision groups: precise (≤15° error), intermediate (>15 and ≤30° error), and imprecise (>30° error).^1^ We predicted pupil size over all timepoints (LME for every 10 ms) based on the precision groups. Random slopes and fixed effects of set size, prototypicality, and their interaction were also added to the model to exclude the possibility that these factors were driving potential pupil effects, using an R formula of Pupil size ∼ Precision group × Set size × Prototypicality + (1 + Set size × Prototypicality|Participant). Pupil size indeed reflected the precision of the encoded material (*p* < 0.05, 480–1410 and 1470–1930 ms from stimulus onset) (Figure 4). More specifically, smaller response errors were associated with stronger orienting responses, as the effect was strongest 600 to 1000 ms after stimulus onset. We also observed the pattern reported in Zhou et al. (2022). The full three-term interaction did not reach significance at any of the timepoints, indicating that the interaction between set size and prototypicality was not modulated by precision (*t* <1.5, *p* > 0.14).

Second, because the pupil orienting response was clearly associated with the precision of encoding, we asked whether pupillary dynamics could capture the depth of encoding on a trial-by-trial basis. To this end, we tested whether baseline pupil size and the pupil orienting response could idiosyncratically predict the precision of the upcoming response on a trial-by-trial basis. Based on the findings from the main analyses, we expected that smaller baseline pupil sizes and stronger pupil orienting responses predict smaller response errors (i.e., more precise encoding). Baseline pupil size and orienting response amplitudes were computed as for the copy task data (median pupil size between –100 and 0 ms and from 500 to 1000 ms from stimulus onset, respectively). These pupil outcomes were used to predict response error on a trial-by-trial basis. We adopted a similar approach as in the analysis over time, as here random slopes and fixed effects of set size, prototypicality, and their interaction were again included in the model, with an R formula of Response error ∼ Baseline pupil size + Orienting response + Set size × Prototypicality + (1 + Set size × Prototypicality|Participant). Higher set sizes strongly predicted larger response errors (β = 5.78 ± 0.52, *t* = 11.08, *p* < 0.001), whereas prototypicality and the interaction term between set size and prototypicality did not reach significance (*t* < 1.15, *p* > 0.25). Supporting and extending our aforementioned findings, we found that baseline pupil size (β = 0.002 ± 0.0008, *t* = 2.22, *p* = 0.026) and the orienting response both predicted VWM precision (β = 0.004 ± 0.0018, *t* = 2.21, *p* = 0.027) on a trial-by-trial basis. More specifically and in line with our hypotheses, smaller baseline pupil sizes and stronger orienting constrictions predicted smaller response errors and thus more precise encoding into VWM.

These reanalyses of the data from Zhou et al. (2022) further support the idea that distinct aspects of attention, as reflected in baseline pupil size and the orienting response, predict the depth of VWM encoding. We show that not only the quantity but also the quality of VWM representations are revealed by pupillary dynamics that underlie distinct aspects of attention.

## Discussion

Until now, VWM encoding depth has often been measured using spatiotemporal properties of gaze, such as gaze position and dwell time, which indicate *where* and *how long* people look (Ballard et al., 1995; Draschkow et al., 2021; Melnik et al., 2018; Somai et al., 2020). Yet, it has remained unclear *how strongly* attention is deployed leading up to and during VWM encoding. Here, we found pupillary dynamics in reanalyzed data (Somai et al., 2020; Zhou et al., 2022) to be linked to VWM encoding depth. More specifically, not only dwell times on to-be-encoded items but also the pupil orienting response and baseline pupil size were predictive of how long people took to copy a pattern of items from VWM, which reflects how much information was encoded into VWM, even within trials. Moreover, we show that not only the amount but also the quality of the representations is reflected in pupillary dynamics.

The pupil orienting response and baseline pupil size are driven by distinct neural underpinnings (reviewed in Strauch et al., 2022). The pupil orienting response is controlled by a superior colliculus (SC)-centered network, which includes the frontal eye fields and anterior cingulate cortex (Strauch et al., 2022). In line with this, microstimulation of SC in primates causes (covert) attentional orienting (Müller, Philiastides, & Newsome, 2005) and changes in pupil size (Wang & Munoz, 2021). Thus, the current results imply that stronger orienting to the to-be-copied stimuli through SC enhances the *depth of sensory processing*, which in turn may boost VWM encoding. Moreover, the current findings show that not only the quantity but also the quality of representations are reflected in the orienting response. In contrast, baseline pupil size is thought to reflect tonic LC firing (Gilzenrat et al., 2010; Jepma & Nieuwenhuis, 2011; Joshi et al., 2016; Naber & Murphy, 2020), which indexes the current arousal state (Strauch et al., 2022). Compatible with the adaptive gain theory (Aston-Jones & Cohen, 2005), we observed that smaller baseline pupil size 100 ms prior to model onset could predict how much was subsequently encoded into VWM on a sample-by-sample basis. This is line with our additional finding that smaller baseline pupil size predicted more precise VWM reports (also see Galeano-Keiner, Pakzad, Brod, & Bunge, 2023). However, as stated earlier, it is complicated to determine “where” any given data point lies along the inverted-U relationship between LC firing rate and task performance. It is therefore possible that in other situations opposite or quadratic relationships are found. Given that baseline pupil size precedes encoding, tonic attentional alerting may reflect how prepared one is to start encoding.

Pupil size can inform about aspects of several, if not all, cognitive processing steps of VWM use, including encoding, maintenance and selection. Our results show that baseline pupil size before encoding already informs how much one will ultimately encode. Previous work demonstrated that, if stimuli differ in brightness, pupil size can reveal how strongly items are encoded (Blom et al., 2016) by leveraging the following phenomenon: The pupil constricts when (covertly) attending bright stimuli and dilates when attending dark stimuli (Binda, Pereverzeva, & Murray, 2014; Hustá, Dalmaijer, Belopolsky, & Mathôt, 2019; Mathôt, Linden, Grainger, & Vitu, 2013; Naber, Alvarez, & Nakayama, 2013). Therefore, a stronger modulation of the pupil light response to the brightness of a given stimulus is linked to deeper encoding of said stimulus (Blom et al., 2016). We add to this by showing that the pupil orienting response shows how strongly one deploys attention, which predicts how much and how *precisely* one encodes more generally, even regardless of brightness.

After encoding, during maintenance, pupil dilation scales with the amount of information stored in (V)WM (Beatty, 1982; Kahneman, 1973; Robison & Unsworth, 2019; Strauch et al., 2022). Recently, Robison and Unsworth (2019) reported larger pupil dilations whenever more items were subsequently correctly answered in a VWM task (but this may not necessarily extend to VWM precision; see Galeano-Keiner et al., 2023).

We hypothesize that the pupil orienting response and these later dilatory components reflect distinct aspects of attention (Strauch et al., 2022). Although it is not unlikely that these components are linked to another to some degree, we posit that orienting reflects the encoding and that the later dilation reveals the amount of maintained information.

Because pupil dilation indicates how much is stored, researchers also investigated whether the pupil could reveal the content of information stored in VWM. Initial attempts that tagged items with distinct brightnesses showed that pupil size does not reveal the overall brightness of retained information (Blom et al., 2016). Although the overall brightness could not be uncovered, pupillometric studies employing retro-cues have investigated whether shifts of internal attention can be tracked. In retro-cue paradigms, participants are cued to internally attend a specific piece of information maintained in VWM (i.e., during maintenance), which shows a robust benefit on VWM task performance (Souza & Oberauer, 2016; van Ede & Nobre, 2023). Shifts of internal attention elicited by retro-cues to bright stimuli constrict the pupil, whereas internally attending dark stimuli cause pupil dilation (Hustá et al., 2019; Zokaei et al., 2019). To summarize, pupil size is a versatile marker of many cognitive processing steps of VWM, reflecting encoding, maintenance, and selection.

When can pupillary dynamics be more informative about VWM encoding depth than dwell time? Although dwell time predicted encoding depth more strongly than pupil size dynamics in our analysis of the data from Somai et al. (2020), this may not always be the case. First, in situations where one’s mind wanders (i.e., blankly staring at the stimuli), pupil size likely outperforms dwell time as a predictor of encoding depth. Dwell time alone cannot capture the disengagement of attention in such situations, but pupillometry provides insights into the ongoing cognitive processes (Unsworth & Robison, 2018). Second, participants do not get the choice of how long they can fixate to-be-encoded stimuli in many existing VWM paradigms, as stimuli are often presented for a limited and fixed time (Van der Stigchel, 2020), as was the case in Zhou et al. (2022). In such cases, dwell time cannot serve as a viable index of VWM encoding, but the orienting response and baseline pupil size could still capture how deeply information is encoded. Third, pupillary dynamics are relatively early signals of encoding depth. In contrast to dwell time, baseline pupil size predicts encoding depth even before stimulus presentation, and the pupil orienting response reveals encoding depth already within ∼700 ms of stimulus onset.

A potential limitation of the current study is that a proxy (namely, build duration) is used for VWM encoding depth. It could be argued that build duration is linked to VWM encoding depth following a negative relationship: More vivid VWM representations could lead to shorter build durations because more vivid representations may make copying the pattern easier. However, a reanalysis of data from a highly similar task (Sahakian et al., 2023) indicates that an increased build duration is strongly linked to more correctly placed items; this positive relationship was found for all participants (see Supplementary Materials). Furthermore, the predictive effects of baseline pupil size and the pupil orienting response on VWM performance were conceptually reproduced and extended in another dataset (Zhou et al., 2022). Thus, build duration serves as a viable index of VWM encoding depth in the current study.

What determines how much one encodes into VWM? In line with previous work (Ballard et al., 1995; Draschkow et al., 2021; Melnik et al., 2018; Sahakian et al., 2023; Somai et al., 2020), the availability of the information in the external world drives this. Of course, how much is encoded ultimately also depends on one's VWM capacity (Luck & Vogel, 2013). Nonetheless, this does not tell the whole story. Our findings show that, driven by separate aspects of attention, spatiotemporal dynamics of gaze and pupil size jointly index how much is ultimately encoded. We posit that how much one encodes into VWM is the integrative outcome of different aspects of attention: one's alertness, how strongly attention is deployed, and how long it is deployed.

## Supplementary Material

Supplement 1

## Footnotes

1 Note that not splitting the data into three groups, but treating response error as a continuous predictor shows conceptually identical results (analysis not reported).
