# Supplementary material for: Differential aspects of attention predict the depth of visual working memory encoding: Evidence from pupillometry
Source: J Vis. 2023 Jun 15;23(6):9. doi: 10.1167/jov.23.6.9 (PMC10278550; doi:10.1167/jov.23.6.9)
Supplement: Supplement 1 [file jovi-23-6-9_s001.pdf]

Supplement to: *Differential Aspects of Attention Predict the Depth of Visual Working Memory Encoding: Evidence from Pupillometry*

1 Build Duration Indexes VWM Encoding Depth

In the data from Somai et al. (2020), intermittent accuracy data regarding item placements were missing. Thus, here we instead used build duration of an index of how much participants encoded on a sample-by-sample basis. To verify that build duration reflects a similar underlying process (i.e. depth of encoding) as the number of correctly placed items, we analyzed open data ($n = 88$) from Sahakian et al. (2023) in which participants performed a highly similar online version of a copy task. Since the way errors were handled between the two tasks (no consequences of mistakes in Somai et al. (2020); in Sahakian et al. (2023) participants were alerted of a mistake and a trial only ended if all items were correctly placed), we only included trials in which no incorrect placements were made. This way, the tasks were as comparable as possible. Next, we computed the average build duration per sample as well as the average number of correct items placed per sample for every trial. These measures were subsequently subjected to a Spearman rank correlation test ($\rho = .89$, $p < .001$), revealing a strong relationship between build duration and the amount of correct items placed (Supplementary Figure 1). Note that a similar result is found when omitting trials in which more than 5 items were correctly placed per sample ($\rho = .80$, $p < .001$) Moreover, we computed this correlation for all participants separately (if they had at least 10 trials

without incorrect placements; $n = 75$), and showed that this positive relationship is highly reliable across participants (one sample t-test: $t(74) = 24.30$, $p < .001$, *Mdn*: $\rho = .81$, range: [.19-.95]).

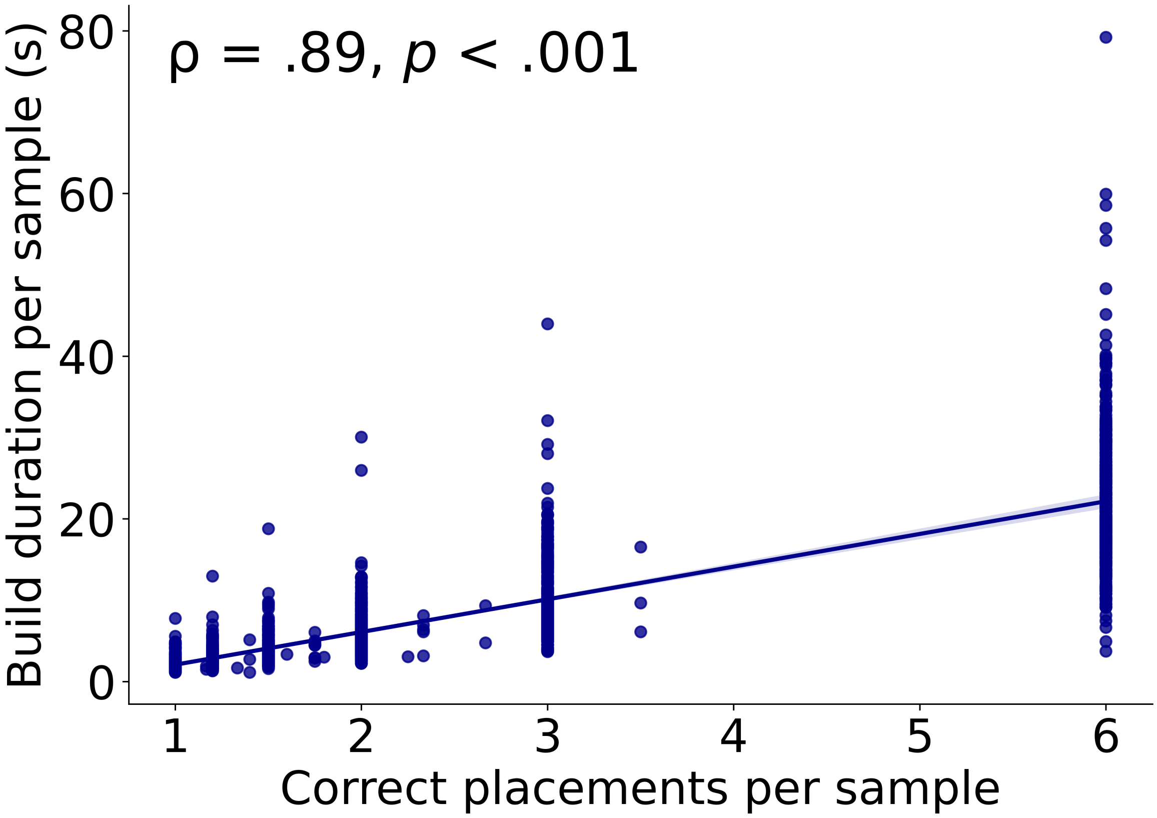

Supplementary Figure 1: Relationship between build duration and the amount of correctly placed items.

2 LME Model Selection

Five models were selected because they were methodologically and theoretically meaningful. In these models we include Condition as a covariate to determine whether potential effects of the ocular metrics could be driven by the delay conditions. Furthermore, all ocular metrics were always accompanied with a interaction term of Experiment to assess potential differences between Experiment 1 and 2. Below are the models that were compared based on complexity and fit:

- m1: Build duration $\sim$ Baseline pupil size x Experiment + Dwell time x Experi-

ment + Orienting response x Experiment + Condition + (1|Participant)

- m2: Build duration ∼ Dwell time x Experiment + Orienting response x Experiment + Condition + (1+Condition|Participant)
- m3: Build duration ∼ Baseline pupil size x Experiment + Dwell time x Experiment + Condition + (1+Condition|Participant)
- m4: Build duration ∼ Baseline pupil size x Experiment + Orienting response x Experiment + Condition + (1+Condition|Participant)
- m5: Build duration ∼ Baseline pupil size x Experiment + Dwell time x Experiment + Orienting response x Experiment + Condition + (1+Condition|Participant)

These models were compared based on their fits using the 'anova' function in R. As stated in the main text, the model that was ultimately chosen was: m5. Table 1 shows that this model, although more complex, also provides a better fit to the data (as tested using the 'anova' function in R; m2 vs. m5: $\chi^2(2) = 8.03$, $p = .018$).

Table 1: Model fit comparisons.

Model	AIC	Log-Likelihood	nPar
m1	5847.3	-2914.7	9
m2	5836.8	-2907.4	11
m3	5844.0	-2911.0	11
m4	5955.3	-2966.7	11
**m5**	**5832.7**	**-2903.4**	**13**

Note. nPar indicates the number of parameters included in the model.

3 Non-Transformed LME Outcomes

For visualization, Figure 3 in the main text shows robust-z scores (see Rousseeuw & Hubert, 2011). We opted to use robust z-scores in the main text since the linear-mixed effects model incorporates a random-effects 'Participant' factor. For completeness, in Supplementary Figure 2 the predictive relationships of orienting amplitude, baseline pupil size and dwell times on build duration are shown using non-transformed values.

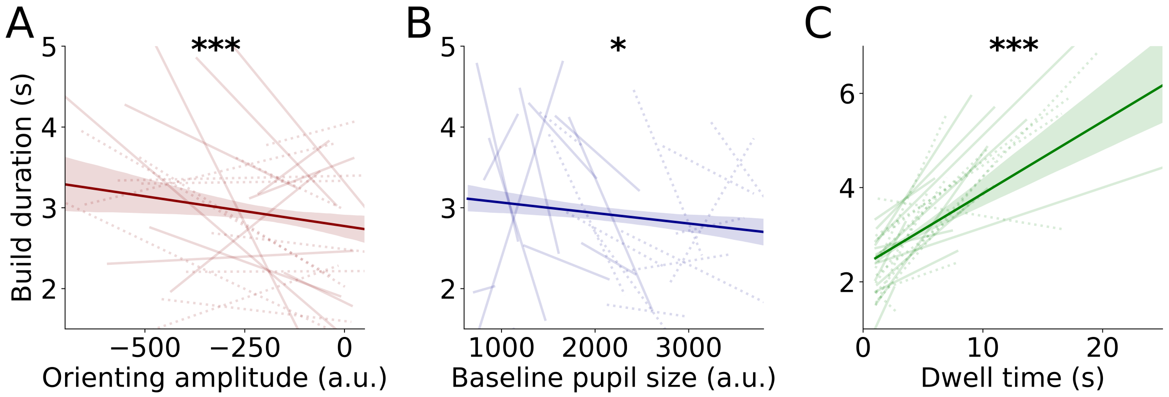

Supplementary Figure 2: Ocular metrics predict encoding depth. A) Pupil orienting amplitude, B) baseline pupil size and C) dwell time predict build duration. Transparent lines are linear regression fits to data per participant. Thick lines show the relationship between the ocular metrics and build duration pooled over all trials (error bars reflect bootstrapped 95% confidence intervals). Transparent lines indicate individual participants. Solid lines and dashed lines indicate Experiments 1 and 2, respectively.

References

Rousseeuw, P. J., & Hubert, M. (2011). Robust statistics for outlier detection. *WIREs Data Mining and Knowledge Discovery*, *1*(1), 73–79. https://doi.org/10.1002/widm.2

Sahakian, A., Gayet, S., Paffen, C. L. E., & Van der Stigchel, S. (2023). Mountains of memory in a sea of uncertainty: Sampling the external world despite useful information in visual working memory. *Cognition*, *234*, 105381. https://doi.org/10.1016/j.cognition.2023.105381

Somai, R. S., Schut, M. J., & Van der Stigchel, S. (2020). Evidence for the world as an external memory: A trade-off between internal and external visual memory storage. *Cortex*, *122*, 108–114. https://doi.org/10.1016/j.cortex.2018.12.017
